# Retraction of: miR-627-3p inhibits osteosarcoma cell proliferation and metastasis by targeting PTN

**DOI:** 10.18632/aging.206128

**Published:** 2024-10-15

**Authors:** Ming He, Peng Shen, Chuang Qiu, Jiashi Wang

**Affiliations:** 1 Department of Orthopedic Surgery, Shengjing Hospital of China Medical University, Shenyang 110004, People’s Republic of China

**Keywords:** PTN, osteosarcoma, miR-627-3p, proliferation, metastasis

**This article has been retracted:** Aging has completed its investigation of this paper. Multiple concerns were raised about this paper, including internal duplications and overlap with unrelated papers from different institutions. The internal duplications were found in Transwell assay images in **Figures 4A–4D** and **5B, 5F**. We also found that the authors reused Transwell assay images and Western blot images from unrelated experiments published earlier in **Figures 4A, 4C, 4D** and **5B** [1, 2] and in **Figures 3G** [3] and **5C** [4]. The external duplications/overlap were with unrelated articles from different institutions, submitted to journals between 2014 and June 2019, before the submission date of the article of interest. These include Transwell assay images in **Figures 4A** [5–7],** 4A, 5F** [8], **4A, 4D** and **5B** [9] as well as Western blot images in **Figures 4F** [10] and** 5G** [11] and duplication of a flow cytometry image in **Figure 3E** [12].

The Scientific Integrity office at Aging contacted the authors, but the authors did not respond to requests to clarify, and the paper was retracted based on an Editorial decision. The Scientific Integrity office also notified the authors’ Institutions about this retraction and added their names to an Editorial Warning list.
